# Cytoplasmic LMO2-LDB1 Complex Activates STAT3 Signaling through Interaction with gp130-JAK in Glioma Stem Cells

**DOI:** 10.3390/cells11132031

**Published:** 2022-06-26

**Authors:** Cheol Gyu Park, Sang-Hun Choi, Seon Yong Lee, Kiyoung Eun, Min Gi Park, Junseok Jang, Hyeon Ju Jeong, Seong Jin Kim, Sohee Jeong, Kanghun Lee, Hyunggee Kim

**Affiliations:** 1 Department of Biotechnology, College of Life Science and Biotechnology, Korea University, Seoul 02841, Korea; lodahpark@gmail.com (C.G.P.); biosanghun05@gmail.com (S.-H.C.); sylee2793@gmail.com (S.Y.L.); eky102@naver.com (K.E.); ak114859@gmail.com (M.G.P.); kkc4152@naver.com (J.J.); guswn9151@naver.com (H.J.J.); wvwv95@hanmail.net (S.J.K.); sohee5003@naver.com (S.J.); biohun3783@naver.com (K.L.); 2 Institute of Animal Molecular Biotechnology, Korea University, Seoul 02841, Korea

**Keywords:** cancer stem cells, glioblastoma, glioma stem cells, LMO2, STAT3

## Abstract

The oncogenic role of nuclear LIM domain only 2 (LMO2) as a transcriptional regulator is well established, but its function in the cytoplasm is largely unknown. Here, we identified LMO2 as a cytoplasmic activator for signal transducer and activator of transcription 3 (STAT3) signaling in glioma stem cells (GSCs) through biochemical and bioinformatics analyses. LMO2 increases STAT3 phosphorylation by interacting with glycoprotein 130 (gp130) and Janus kinases (JAKs). LMO2-driven activation of STAT3 signaling requires the LDB1 protein and leads to increased expression of an inhibitor of differentiation 1 (ID1), a master regulator of cancer stemness. Our findings indicate that the cytoplasmic LMO2-LDB1 complex plays a crucial role in the activation of the GSC signaling cascade via interaction with gp130 and JAK1/2. Thus, LMO2-LDB1 is a *bona fide* oncogenic protein complex that activates either the JAK-STAT signaling cascade in the cytoplasm or direct transcriptional regulation in the nucleus.

## 1. Introduction

Glioblastoma (GBM) is a catastrophic primary brain tumor [1]. Despite conventional therapy for patients with GBM, the median survival is approximately 15 months [2,3].

Cancer stem cells (CSCs) possess the characteristics of normal stem cells (NSCs), such as self-renewal, differentiation potential, and unique niche [4,5,6,7]. Numerous studies have supported the existence of CSCs in various types of tumors, such as leukemia, breast cancer, and GBM [8,9,10]. CSCs are responsible for tumor propagation, resistance to chemoradiotherapy, and tumor recurrence [11,12,13]. Numerous studies have demonstrated that CSCs maintain their cancer stemness through a variety of ligand-induced receptor signaling pathways, such as Notch, Wnt, Hedgehog, NF-κB, JAK-STAT, and TGF-β [14]. However, among the molecular mechanisms regulating the properties of CSCs, cell-intrinsic signaling pathways independent of extracellular signaling ligands are relatively less documented.

LIM domain only 2 (LMO2) consists of two LIM (LIN-11, Isl1, and MEC-3) domains—LIM1 and LIM2. LIM domains are composed of two zinc-finger binding motifs that mediate interactions with other proteins [15]. LMO2 proteins act as bridging molecules for the formation of the transcriptional complex with LIM domain-binding 1 (LDB1), TAL1, and LYL1 (class II basic helix-loop-helix (bHLH) transcription factors), TCF3 (a class I bHLH transcription factor), and GATA1 (a zinc finger transcription factor) [16,17,18]. The LMO2-mediated transcriptional complex is an essential transcriptional regulator of hematopoiesis and angiogenesis during early development [19,20]. In the case of cancers, aberrant expression of LMO2 induces tumor aggressiveness and CSC characteristics in T-ALL and GBM [21,22]. In contrast, some studies have shown that LMO2 is a favorable prognostic marker in pancreatic cancer and diffuse large B-cell lymphoma [23,24]. Thus, it is necessary to elucidate the proteins that interact with LMO2 to elicit its oncogenic function. Most studies have focused on the transcriptional role of LMO2 within the nucleus, whereas few studies have shown the presence of cytoplasmic LMO2 in vitro and in vivo, suggesting the potential tumor-suppressive functions of LMO2 in the cytoplasmic compartment by suppressing Wnt signaling via interaction with DVL-1/2 [25]. Conversely, little is known about whether LMO2 has an oncogenic function by interacting with cytoplasmic proteins. Therefore, the aim of this study was to investigate the oncogenic role of cytoplasmic LMO2 in glioma stem cells (GSCs) and to elucidate the signaling mechanisms induced through complex with proteins that bind to cytoplasmic LMO2.

## 2. Materials and Methods

### 2.1. Cell Lines and Culture Conditions

The human glioblastoma stem cell lines (GSC11 and GSC20) were kindly provided by Dr. Erick P. Sulman (Department of Radiation Oncology, The University of Texas MD Anderson Cancer Center, Houston, TX, USA). These cell lines were cultured using Dulbecco’s modified Eagle’s medium/F12 (DMEM/F12) (HyClone, Logan, UT, USA) supplemented with 0.2% B27 (Invitrogen, Carlsbad, CA, USA), 20 ng/mL epidermal growth factor (EGF) (R&D Systems, Minneapolis, MN, USA), 20 ng/mL basic fibroblast growth factor (bFGF; R&D Systems, Minneapolis, MN, USA), 1% penicillin or streptomycin (HyClone, Logan, UT, USA), 2 mmol/L L-glutamine (HyClone, Logan, UT, USA), and 50 μg/mL gentamicin (Cellgro, Corning, NY, USA). The human B cell lymphoma cell line, JM1, was purchased from ATCC (ATCC, Manassas, VA, USA), and the human erythroleukemia cell line TF-1 was purchased from the ATCC. These cells were cultured in RPMI1640 (HyClone, Logan, UT, USA) supplemented with 10% fetal bovine serum (HyClone, Logan, UT, USA), 1% penicillin or streptomycin, 2 mM L-glutamine, and 50 µg/mL gentamicin. TF-1 cells were cultured in medium containing 2 ng/mL GM-CSF (R&D Systems, Minneapolis, MN, USA).

The human glioma cell lines (U87MG and U373MG) were purchased from the ATCC (ATCC, Manassas, VA, USA). The HEK293FT cell line was purchased from Thermo Fisher Scientific (Thermo Fisher Scientific, Waltham, MA, USA). These cells were cultured in DMEM with high glucose (4500 mg/L) supplemented with 10% fetal bovine serum (HyClone, Logan, UT, USA), 1% penicillin/streptomycin, 2 mM L-glutamine, and 50 µg/mL gentamicin.

### 2.2. Plasmids

HA-LMO2, LIM1-GFP, LIM2-GFP, FLAG-FERM, FLAG-SH2, FLAG-JH2, FLAG-JH1, and FLAG-gp130^ID^ were inserted into the pcDNA 3.1(+)-Puro. The cDNA of JAK1, JAK2, and gp130 was generously gifted by Dr. Hong-Hee Kim (Seoul National University, Republic of Korea), Dr. Joohun Ha (Kyunghee University, Republic of Korea), and Dr. Gerhard Müller-Newen (RWTH Aachen University, Germany). FLAG-JAK1, FLAG-JAK2, and FLAG-gp130 were cloned into the pCMV-TAG2B. Human LMO2 and HA-FLAG-LMO2 were cloned using the pLL-CMV-puro. 3× SBE (STAT3 binding element)-mCMV-EGFP was cloned into the pCDH-CMV-puro vector. The pCDH-CMV-gp130-DsRED was established for fluorescence-activated cell sorting (FACS). Short hairpin RNAs (shRNAs) targeting human LMO2 were purchased from Sigma. The shID1 plasmid was kindly provided by Dr. Xun Jin (Lerner Research Institute, USA).

### 2.3. Lentiviral Transduction and Gene Transfections

Each shRNA vector was transfected with the second-generation lentiviral packaging plasmids Δ8.9 and VSV-G to produce the lentivirus using the LipoJet transfection reagent (SignaGen Laboratories, Frederick, MD, USA) into transformed HEK293FT cells. Non-targeting shRNA (pLKO.1 shNT Puro, Addgene, Watertown, MA, USA) was used as a control. The culture medium was harvested 24 h after transfection, incubated with a Lenti-X concentrator (Clontech, Kusatsu, JPN), and centrifuged to obtain the concentrated lentivirus. Finally, the cells were infected with the lentiviruses in the presence of 6 μg/mL hexadimethrine bromide (Sigma, Missoula, MO, USA) for 24 h.

LDB1 siRNA was purchased from Sigma, and the ScreenFect A transfection reagent (Wako Pure Chemical, Osaka, JPN) was used according to the manufacturer’s instructions. TF-1 cells were infected with the lentivirus using the spinoculation method; then, 2 × 10^5^ TF-1 cells were plated in 6-well plates with fresh media containing 6 μg/mL hexadimethrine bromide and lentivirus. Subsequently, these cells were centrifuged at 2000× *g* for 2 h at room temperature. The next day, the lentivirus was removed, and a fresh medium with GM-CSF (R&D Systems, Minneapolis, MN, USA) was added.

### 2.4. RNA Extraction, Quantitative Reverse Transcription-PCR

By following the manufacturer’s instructions, total RNA was obtained from cells using QIAzol lysis reagent (QIAGEN, Hilden, DEU), and DNase I-treated RNA (1 μg) was reverse-transcribed into cDNA using a RevertAid first-strand cDNA synthesis kit (Thermo Fisher Scientific, Waltham, MA, USA). The qRT-PCR reaction was utilized using the CFX Connect Real-Time PCR Detection System (Bio-Rad, Hercules, CA, USA) using TB Green Premix Taq (Takara Bio Inc., Kusatsu, JPN). Gene expression was quantified using the standard 2−∆∆Ct method as previously described [26]. The mRNA expression level of each target gene was normalized to that of the GAPDH control.

### 2.5. Co-Immunoprecipitation and Western Blot Assay

For the Co-IP assay, the previously described plasmids were co-transfected into HEK293FT cells, and the cells were extracted using IP lysis buffer (Thermo Fisher Scientific, Waltham, MA, USA) supplemented with 1 mM PMSF (Sigma, Missoula, MO, USA), a protease inhibitor (Roche, Basel, CHE), and phosphatase inhibitors (2.5 mM Na_4_P_2_O_7_, 1 mM NaF, and 1 mM Na_3_VO_4_). The lysates were pre-cleared with Protein A/G agarose (Thermo Fisher Scientific, Waltham, MA, USA), and the proteins (500–1000 µg) were precipitated using the anti-FLAG antibody (1:100; Sigma, Missoula, MO, USA), anti-HA antibody (1:100; Cell Signaling, Danvers, MA, USA), and Protein A/G agarose. The FLAG or HA-binding proteins were washed with IP lysis buffer and eluted with 1× LDS sample buffer (Invitrogen, Carlsbad, CA, USA) for 3 min at 100 °C. SDS-PAGE was used to separate the eluted proteins, which were then transferred to a poly-vinylidene fluoride membrane (Millipore, Darmstadt, DEU). The membranes were blocked with 5% non-fat milk and incubated with primary antibodies. Following washing, the membranes were incubated with a horseradish peroxidase-conjugated anti-IgG secondary antibody (Thermo Fisher Scientific, Waltham, MA, USA) and visualized using PicoEPD Western Reagent (Elpis Biotech, Daejeon, KOR). Primary antibodies were used at the following dilutions: anti-FLAG (1:1000, Sigma, Missoula, MO, USA) and anti-HA (1:1000, Sigma, Missoula, MO, USA).

For the endogenous Co-IP assay, proteins of glioblastoma stem cells, GSC11 and GSC20, were extracted using IP lysis buffer supplemented with 1 mM PMSF, protease inhibitor, and phosphatase inhibitors. The lysates were pre-cleared with Protein A/G agarose, and lysate (1 mg) was precipitated using the anti-LMO2 antibody (4 µg, R&D systems, Minneapolis, MN, USA), anti-LDB1 antibody (4 µg, Abcam, Cambridge, GBR), anti-JAK1 antibody (4 µg, Cell Signaling, Danvers, MA, USA), and anti-GP130 antibody (4 µg, Abcam, Cambridge, GBR). The precipitated proteins were washed with IP lysis buffer and PBS containing 1 mM PMSF.

Cell extracts were lysed by sonication in RIPA lysis buffer (LPS Solution, Daejeon, KOR) containing 1 mM β-glycerophosphate, 2.5 mM Na_4_P_2_O_7_, 1 mM NaF, 1 mM Na_3_VO_4_, and protease inhibitor. The protein concentration was determined using the Bradford reagent (Bio-Rad, Hercules, CA, USA), following the manufacturer’s instructions. Approximately 10–50 µg of proteins were resolved by SDS-PAGE, and immunoblotting was performed as described above. The primary antibodies used were as follows: anti-LMO2 (1:500), anti-LDB1 (1:500), anti-JAK1 (1:500), anti-JAK2 (1:500), anti-gp130 (1:2000), anti-β-actin (1:10,000, Santa Cruz Biotechnology, Dallas, TX, USA), anti-pY705-STAT3 (1:500, Cell Signaling, Danvers, MA, USA), anti-STAT3 (1:1000, Cell Signaling, Danvers, MA, USA), and anti-ID1 (1:500, Biocheck, South San Francisco, CA, USA). β-Actin was used as a loading control.

### 2.6. Promoter-Luciferase Reporter Assay

Using the Dual-Glo Luciferase Assay System (Promega, Madison, WI, USA), the transcriptional activity of ID1 was measured by analyzing the relative luciferase activities of the pGL3-ID1 promoter and pTOP or FOP-Flash. According to the manufacturer’s instruction, transfection efficiency was normalized with co-transfected Renilla luciferase activity.

### 2.7. In vitro Limiting Dilution Sphere Formation Assay

In 96-well plates containing DMEM/F12 with B27, EGF, and bFGF, decreasing numbers of cells (20, 10, 5, 2, and 1) were seeded in individual wells for an in vitro limiting dilution assay (*n* = 18). After 14 days, light microscopy was used to count spheres larger than 10 µm in diameter. The frequency of stem cells was calculated using the ELDA software (Parkville Victoria, AUS), which is available at https://bioinf.wehi.edu.au/software/elda (accessed on 10 November 2018).

### 2.8. Migration Assay

For the migration assay, 5.0 × 10^3^ U87MG cells were seeded and incubated in ultra-low attachment 96-well plates for 72 h. After incubation, the spheroids were harvested and immediately seeded on a matrigel (BD Biosciences, Franklin Lakes, NJ, USA) -coated 6-well plates. Each well was scanned at 3-h intervals, and the sprouting area and length from the center of the spheroid were measured and quantified using IncuCyte Zoom (Sartorius, Goettingen, DEU, Version; 2016B).

### 2.9. Proximity Ligation Assay

Glioblastoma cells were attached to Matrigel-coated 48-well slides to perform the proximity ligation assay, then the cells on the slides were fixed with 4% paraformaldehyde. The fixed cells were permeabilized using 0.5% Triton ×-100 for 30 min. Samples were stained using primary antibody followed by Duolink in situ PLA probes with anti-goat PLUS, anti-rabbit MINUS, and Duolink in situ detection reagents Red (Sigma, Missoula, MO, USA) following the manufacturer’s instructions. anti-LMO2 (1:200), anti-LDB1 (1:200), anti-JAK1 (1:200), anti-JAK2 (1:200), and anti-GP130 (1:500) were used as primary antibodies. Samples were incubated at 4 °C for 16 h with primary antibodies and counterstained with the nuclear dye 4′,6-diamidino-2-phenylindole (1 µg/mL, Sigma, Missoula, MO, USA) for 5 min. Samples were imaged using a confocal laser-scanning microscope LSM800 after staining (Carl-Zeiss; Plan-Apochromat ×63/1.40 Oil DIC M27, Jena, DEU).

### 2.10. Liquid Chromatography with Tandem Mass Spectrometry (LC-MS/MS)

Protein bands of interest were excised and digested in-gel with sequencing grade modified trypsin (Promega, Madison, WI, USA) for in-gel protein digestion. In brief, each protein spot was excised from the gel, placed in a polypropylene (Eppendorf, Hamburg, DEU) tube, and washed four to five times (until the gel was clear) with 150 µL of 1:1 acetonitrile/25 mM ammonium bicarbonate, pH 7.8. The gel slices were dried in a Speedvac concentrator and then rehydrated in 30 µL of 25 mM ammonium bicarbonate, pH 7.8, containing 20 ng of trypsin. After incubation at 37 °C for 20 h, the liquid was transferred to a new tube. Tryptic peptides remaining in the gel matrix were extracted for 40 min at 30 °C with 20 µL of 50% (*v*/*v*) aqueous acetonitrile containing 0.1% (*v*/*v*) formic acid. The combined supernatants were evaporated in a Speedvac concentrator and dissolved in 8 µL of 5% (*v*/*v*) aqueous acetonitrile solution containing 0.1% (*v*/*v*) formic acid for mass spectrometric analysis.

The resulting tryptic peptides were separated and analyzed using reversed phase capillary HPLC directly coupled to a Finnigan LCQ ion trap mass spectrometer (LC-MS/MS) to identify proteins by LC-MS/MS. 0.1 × 20 mm trapping and a 0.075 × 130 mm resolving column were packed with Vydac 218MS low trifluoroacetic acid C18 beads (5 µm in size, 300 Å in pore size; Vydac, Hesperia, CA, USA) and placed in-line. The peptides were bound to the trapping column for 10 min with 5% (*v*/*v*) aqueous acetonitrile containing 0.1% (*v*/*v*) formic acid; then the bound peptides were eluted with a 50 min gradient of 5%–80% (*v*/*v*) acetonitrile containing 0.1% (*v*/*v*) formic acid at a flow rate of 0.2 µL/min. For tandem mass spectrometry, the full mass scan range mode was m/z = 450–2000 Da. After determination of the charge states of an ion on zoom scans, product ion spectra were acquired in MS/MS mode with a relative collision energy of 55%.

The individual spectra from MS/MS were processed using the TurboSEQUEST software (Thermo Quest, San Jose, CA, USA). The generated peak list files were used to query either the MSDB database or SwissProt using the MASCOT program (http://www.matrixscience.com, accessed on 19 September 2018). Modifications of methionine and cysteine, peptide mass tolerance at 2 Da, MS/MS ion mass tolerance at 0.8 Da, allowance of missed cleavage at 2, and charge states (+1, +2, and +3) were taken into account. Only significant hits, as defined by MASCOT probability analysis, were considered initially.

The mass spectrometry proteomics data have been deposited to the ProteomeXchange Consortium via the PRIDE partner repository with the dataset identifier PXD028254 [27].

### 2.11. Bioinformatics Analysis

RNA-Seq was performed at the Beijing Genomics Institute (BGI). LMO2 RNA-seq data were grouped based on >2-fold change in expression with >0.7 probability value to establish the LMO2 signature (GEO: GSE182169)**.** Gene set enrichment analysis (GSEA) was conducted using 3.0 version GSEA (Broad Institute) and the RNA sequencing data set, which includes triplicate FPKM values for each gene. The results were considered statistically significant with a false discovery rate (FDR) < 0.25. Single-sample GSEA (ssGSEA) was analyzed with 8.0 version ssGSEAProjection (https://cloud.genepattern.org, accessed on 5 October 2019). Normalized gene expression values and enrichment scores were calculated to z-scores. The extent of correlation is displayed as a correlation coefficient (r).

The ssGSEA was conducted using LMO2 (or LMO4)-signature and STAT3-signature to evaluate the clinical significance of LMO2 (or LMO4) and STAT3 signaling. Next, the group was divided into two groups (Group 1: STAT3-signature-High LMO2 (or LMO4)-signature-High; Group 2: STAT3-signature-Low and LMO2 (or LMO4)-signature-Low) based on the following criteria: signature-high = greater than the mean of the ssGSEA score + standard deviation, signature-Low = less than the mean of the ssGSEA score–standard deviation. Patient prognosis between the two groups was then compared.

The ID1 RNA-seq data performed by eBiogen were grouped based on >2-fold change in expression to establish ID1 signature as determined by comparing the mean expression values (Student’s *t*-test; *p* < 0.05) (GEO: GSE182670).

We identified 689 glioblastoma-related oncogenes in the literature by performing literature-based mining using Beegle (http://beegle.esat.kuleuven.be/, accessed on 20 June 2022) [28].

Predicted transcription factor binding sites were visualized using ConTra v3 (http://bioit2.irc.ugent.be/contra/v3/, accessed on 20 June 2022) [29]. In the first step, the human ID1 gene was selected for exploration to analyze the promoter region (2200-bp upstream). Next, we selected STAT3 (TRANSFAC20113, JASPAR_CORE_2016) for visualization with a core and similarity matrix stringency of 0.9 and 0.75, respectively.

The Repository of Molecular Brain Neoplasia Data (REMBRANDT) and The Cancer Genome Atlas Program (TCGA) database were used to analyze gene expression, survival, and correlation between assigned groups in various tumor patients [30]. The REMBRANDT data were obtained from the GlioVis data portal (http://gliovis.bioinfo.cnio.es/, accessed on 20 June 2022) [31]. TCGA data were obtained from the Firebrowse data portal (http://firebrowse.org, accessed on 20 June 2022). Chinese Glioma Genome Atlas (CGGA) data were obtained from the CGGA database (http://cgga.org.cn, accessed on 20 June 2022)

### 2.12. Quantification and Statistical Analysis

The statistical significance of data was analyzed using Student’s *t*-test in the paired groups. Data are expressed as the mean ± standard deviation (SD). *p* < 0.05 (*), *p* < 0.01 (**), and *p* < 0.001 (***).

## 3. Results

### 3.1. LMO2 Regulates STAT3 Activity in GSCs

**Figure 1:**
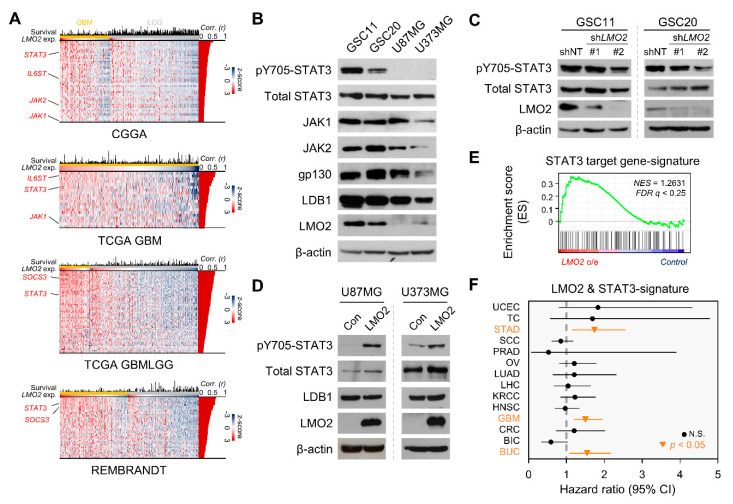
The LMO2 Activates STAT3 Signaling in Glioma Stem Cells (GSCs) (**A**) The heatmap shows genes that are positively correlated with LMO2 in the patient-meta dataset (CGGA; Chinese glioma genomics atlas, *n* = 692; TCGA GBM; the cancer genome atlas glioblastoma, *n* = 538; TCGA LGG; TCGA low-grade glioma, *n* = 667; REMBRANDT; the repository of molecular brain neoplasia data, *n* = 444) Pearson correlation coefficient values (*Corr (r)*) were analyzed from mRNA levels between *LMO2* and STAT3-related genes, presented at the right position of each heatmap. (**B**) Cell lysates from GSCs and non-GSCs were immunoblotted with antibodies specific for pY705-STAT3, total STAT3, JAK1, JAK2, GP130, LDB1, LMO2, and β-actin. (**C**) Cell lysates of GSCs transduced with either LMO2 shRNA- or non-target shRNA-expressing lentiviruses were immunoblotted with antibodies specific for pY705-STAT3, total STAT3, LMO2, and β-actin. (**D**) Cell lysates of non-GSCs (U87MG and U373MG) expressing control or LMO2 proteins were immunoblotted with antibodies specific for pY705-pSTAT3, total STAT3, LDB1, LMO2, and β-actin. (**E**) Gene set enrichment analysis (GSEA) showed the STAT3 target gene signature enrichment in U87MG-LMO2 cells. (**F**) Hazard ratio analysis using LMO2 & STAT3-signature in the pan-cancer dataset. Horizontal bars represent 95% CIs of hazard ratios. Orange lines and dots represent tumors with statistically significant hazard ratios. (UCEC; uterine corpus endometrial carcinoma, TC; thyroid carcinoma, STAD; stomach adeno-carcinoma, SCC; skin cutaneous carcinoma, PRAD; prostate adenocarcinoma, OV; ovarian serous cystadenocarcinoma, LUAD; lung adenocarcinoma, LHC; liver hepatocellular carcinoma, KRCC; kidney renal clear cell carcinoma, HNSC; head and neck squamous cell carcinoma, GBM; glio-blastoma, CRC; colorectal adenocarcinoma, BIC; breast invasive carcinoma, BUC; bladder urothelial carcinoma)

Previously, we have shown that the expression of LMO2 is upregulated in glioma stem cells (GSCs) [22]. Single-sample gene set enrichment analysis (ssGSEA) data showed that LMO2 was positively correlated with GSC-related transcription factors in The Cancer Genome Atlas (TCGA) GBM and cancer cell line encyclopedia database [32,33] (Supplementary Figure S1A). We first performed bioinformatics analysis using GSEA in patients with GBM to elucidate the mechanism regulating GSC properties by LMO2. We then found an enriched pattern of STAT3 target gene-signature in LMO2-High patients (Supplementary Figure S1B). In addition, these results were consistent with the analysis based on the patient-meta dataset, such as the Chinese Glioma Genomics Atlas, TCGA, and REMBRANDT (Figure 1A).

We hypothesized that STAT3 activity is differentially regulated between GBM cells and GSCs, depending on LMO2 expression levels. Immunoblot analysis showed that phosphorylated STAT3 at the 705 tyrosine residue (pY705-STAT3) and that LMO2 was highly expressed in GSCs (GSC11 and GSC20) but not in non-GSCs (U87MG and U373MG) (Figure 1B); a similar trend was also observed at the mRNA level (Supplementary Figure S1C). To determine whether the presence of LMO2 is required for the activation of STAT3 in GSCs and GBM cells, we depleted LMO2 expression levels by transducing an LMO2-specific small hairpin RNA (shRNA) lentiviral vector and found that phosphorylated STAT3 was decreased by LMO2 depletion in GSCs (Figure 1C). In contrast, overexpression of LMO2 in non-GSCs resulted in an increase in STAT3 phosphorylation at the 705 tyrosine residue (Figure 1D). In addition, GSEA showed that LMO2-overexpressing U87MG (U87MG-LMO2) cells exhibited an enrichment of the STAT3 target gene signature (Figure 1E). In the clinical data, the LMO2 expression level and pY705-STAT3 reverse-phase protein array score were positively correlated in the TCGA GBMLGG (Low-grade glioma) dataset (Supplementary Figure S1D).

To evaluate the clinical significance of LMO2 and STAT3 signaling, we performed pan-cancer survival analysis with LMO2- & STAT3-signature in 14 tumor types from TCGA [34]. The high LMO2 & STAT3-signature had a prognostic value in three tumor types, namely, stomach adenocarcinoma (STAD), GBM, and urothelial bladder carcinoma (BUC), and had a positive correlation between LMO2-signature and STAT3-signature in these tumors (Figure 1F, Supplementary Figure S1E), (UCEC; uterine corpus endometrial carcinoma, TC; thyroid carcinoma, SCC; skin cutaneous carcinoma, PRAD; prostate adenocarcinoma, OV; ovarian serous cystadenocarcinoma, LUAD; lung adenocarcinoma, LHC; liver hepatocellular carcinoma, KRCC; kidney renal clear cell carcinoma, HNSC; head and neck squamous cell carcinoma, CRC; colorectal adenocarcinoma, BIC; breast invasive carcinoma). To determine whether the direct interaction of LMO2 with STAT3 is related to the transcriptional activity of STAT3, we performed co-immunoprecipitation (Co-IP) experiments using an anti-LMO2 antibody. The results showed that endogenous LMO2 did not co-precipitate STAT3 in GSCs (Supplementary Figure S1F). In addition, Co-IP analysis with anti-HA antibody showed that exogenous LMO2 did not bind to STAT3 in HEK293FT cells expressing HA-LMO2 (Supplementary Figure S1G). Taken together, our results showed that a correlation between LMO2 expression and STAT3 signaling was specifically observed in GSCs, but there was no physical interaction between LMO2 and STAT3.

### 3.2. Cytoplasmic LMO2-LDB1 Complex Regulates STAT3 Activity in GSCs

**Figure 2:**
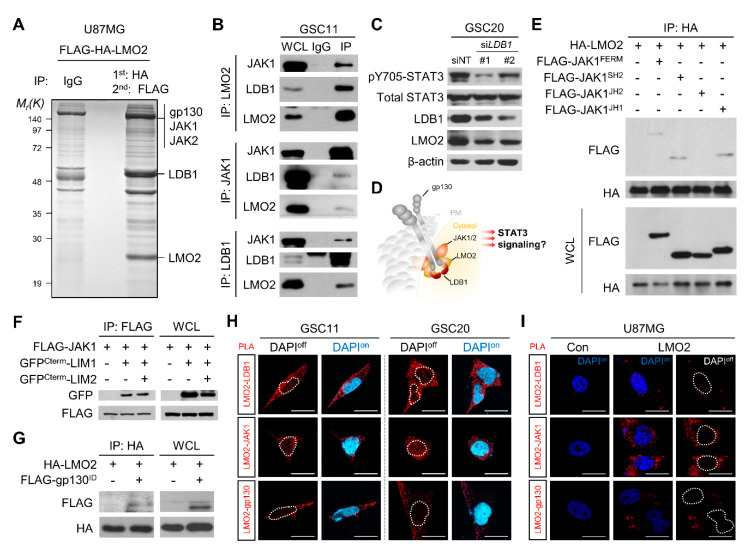
The LMO2-LDB1 Complex Regulates STAT3 Activity via Interaction with gp130 and JAKs. (**A**) Coomassie Brilliant Blue staining of affinity-purified protein from HA-FLAG-LMO2-expressing U87MG cells. LMO2-interacting protein bands were analyzed by LC-MS/MS. (**B**) Co-immunoprecipitation (Co-IP) of LMO2, JAK1, and gp130 in GSC11 cells IgG is the control antibody for Co-IP. Whole cell lysates (WCLs, lane 1) or immunoprecipitates generated with the indicated antibodies (lane 3) or a control IgG antibody (lane 2) were immunoblotted with the indicated antibodies. (**C**) Cell lysates of GSC20 cells transfected with either non-target siRNA or si*LDB1* were immunoblotted with antibodies specific for pY705-pSTAT3, total STAT3, LDB1, LMO2, and β-actin. (**D**)A schematic diagram showing that LMO2 binds to LDB1, JAK1/2, and gp130. (**E**) A Co-IP experiment to identify the LMO2-interacting JAK1 domains (FERM, SH2, JH2, and JH1) in HEK293FT cells. HEK293FT cells were transfected with expression vectors encoding FLAG-JAK1^FERM^, FLAG- JAK1^SH2^, FLAG-JAK1^JH1^, and FLAG-JAK1^JH2^ together with HA-LMO2. The cell lysates were immunoprecipitated with an antibody against HA captured with agarose A/G beads and blotted using anti-FLAG and anti-HA antibodies. The expression levels were checked using 40 μg of WCLs and immunoblotted with the indicated antibodies. (**F**) A Co-IP experiment to identify the LMO2 domains that bind to JAK1 proteins in HEK293FT cells. HEK293FT cells were transfected with expression vectors encoding GFP^Cterm^-LIM1 or GFP^Cterm^-LIM2 together with FLAG-JAK1 (GFP^Cterm^ means GFP-encoding DNA sequence is fused with the C-terminus region of the gene of interest) The cell lysates were immunoprecipitated with an antibody against FLAG captured with agarose A/G beads and blotted using anti-GFP and anti-FLAG antibodies. The expression levels were checked using 40 μg of WCLs and immunoblotted with the indicated antibodies. (**G**) A Co-IP experiment was performed to identify whether the gp130 intracellular domains (ID) interact with LMO2 in HEK293FT cells. HEK293FT cells were transfected with an expression vector encoding FLAG-gp130^ID^ and HA-LMO2. The cell lysates were immunoprecipitated with an antibody against HA captured with agarose A/G beads and blotted using anti-FLAG and anti-HA antibodies. The expression levels were checked using 40 µg of WCLs and immunoblotted with the indicated antibodies. (**H**) Proximity ligation assay in GSC11 and GSC20 cells. The cells were stained with the indicated antibodies. Representative images are presented. Nuclei were stained with DAPI. Red dots indicate the protein interaction signals. Scale bars: 20 µm. (**I**) Proximity ligation assay in U87MG cells expressing control or LMO2 proteins. The cells were stained with the indicated antibodies. Representative images are presented. Nuclei were stained with DAPI. Red dots indicate the protein interaction signals. Scale bars: 20 µm. See also Supplementary Figure S2C.

The phosphorylation of STAT3 was affected by LMO2 expression, indicating that the upstream effectors of STAT3 signaling could be regulated by LMO2 (Figure 1C,D). However, we did not observe a physical interaction between LMO2 and STAT3 (Supplementary Figure S1F,G). It was previously shown that LMO2 interacts with the Wnt signaling effector DVL-1/2 and attenuates the Wnt pathway in breast and colorectal cancers [25]. Moreover, the transcriptional roles of LMO2 vary depending on which transcription factor LMO2 binds to [35,36,37]. Therefore, we hypothesized that LMO2 activates STAT3 signaling via its interaction with the upstream regulator of STAT3. First, to identify the binding partner of LMO2 in STAT3 activation, we isolated LMO2 binding proteins from GBM cells. Cell extracts from U87MG cells stably expressing LMO2 containing tandem N-terminal HA and Flag epitopes were analyzed using tandem affinity purification. Thereafter, the affinity-purified LMO2 binding proteins were analyzed by liquid chromatography-tandem mass spectrometry (LC-MS/MS). We identified several STAT3 upstream effectors, such as gp130, JAK1, JAK2, and the well-known LMO2 binding partner, LDB1 [16] (Figure 2A). To confirm the interaction between LMO2 and its binding partners, we transfected HEK293FT cells with FLAG-tagged JAK1, JAK2, or gp130 in the presence of HA-tagged LMO2 and performed Co-IP assays. We found that all three STAT3 upstream effectors were physically bound to LMO2 (Supplementary Figure S2A). We performed Co-IP experiments to determine whether endogenous LMO2 could interact with STAT3 upstream effectors in GSCs or non-GSCs. The results showed that the interaction of LMO2 with the STAT3 upstream effectors was observed in GSCs but not in non-GSCs (Figure 2B, Supplementary Figure S2B). Among LMO2 binding proteins in Co-IP data, LDB1 is known to act as a molecular bridge that interacts with LMO2 and several transcription factors [38]. However, it is not known whether the binding of LMO2 to LDB1 is required for binding to STAT3 upstream effectors (Figure S1H). Therefore, we examined the role of LDB1 in regulating LMO2-driven STAT3 activity by small interfering RNA (siRNA)-mediated *LDB1* depletion. The results showed that *LDB1* depletion led to a decrease in pY705-STAT3 levels (Figure 2C).

Next, we investigated the binding sites of each protein involved in the formation of a hypothetical multiprotein complex (Figure 2D). To identify the JAK1 domains involved in interacting with LMO2, we developed constructs encoding different domains of JAK1. The FERM, SH2-like, and JH1 domains showed a binding affinity for LMO2; however, the JH2 domain did not interact with LMO2 (Figure 2E). LMO2 is composed of two LIM domains, each comprising two zinc fingers of similar length and homology. To determine which LIM domain-mediated JAK binding, we constructed GFP fusion proteins containing the first LIM domain (LIM1) and second LIM domain (LIM2) and found that both LIM1 and LIM2 showed equal binding affinity to JAK1 (Figure 2F). We further evaluated whether gp130 interacts with LMO2 through its intracellular region and found that LMO2 binds to the intracellular domain of gp130 (Figure 2G).

Because, traditionally, LMO2 functions as a transcription regulator in the nucleus, we determined the cellular compartment where LMO2 and STAT3 upstream effectors interacted using the proximity ligation assay (PLA) in GSCs and non-GSCs. LMO2-LDB1 or STAT3 upstream effectors interacted in the cytoplasm of GSCs, but not in GBM cells (Figure 2H, Supplementary Figure S2C). Next, we investigated whether LMO2 is a key protein that regulates complex formation with STAT3 upstream effectors. In the U87MG cells, the expression of proteins (JAK1, JAK2, gp130, and LDB1) other than LMO2 was similar to that of GSCs. Therefore, we overexpressed LMO2 in U87MG cells and found that the interaction of LMO2-gp130 and LMO2-JAK1 was observed in U87MG-LMO2 cells but not in control cells, as determined by PLA (Figure 1B and Figure 2I). We also found that activation of STAT3 by LMO2 overexpression was diminished by siRNA-mediated LDB1 depletion in these cells (Supplementary Figure S2D). These results indicate that the cytoplasmic LMO2-LDB1 complex is required for STAT3 activation by interacting with STAT3 upstream effectors. We hypothesized that LMO2 exhibits cytoplasmic function only when all components of cytoplasmic are equipped. Thus, we investigated whether ectopic expression of gp130 promotes LMO2-driven STAT3 activity in TF-1 leukemia cells, which highly express other complex proteins except for gp130 (Supplementary Figure S2E). The results showed that gp130 overexpression in TF-1 cells resulted in a marked increase in pY705-STAT3 levels (Supplementary Figure S2F). These results suggest that interaction of the LMO2-LDB1 complex with STAT3 upstream effectors occurs in the cytoplasm and induces STAT3 phosphorylation.

### 3.3. Level of LMO2 and gp130 Is Critical for STAT3 Activity in Heterogeneous GSCs

**Figure 3:**
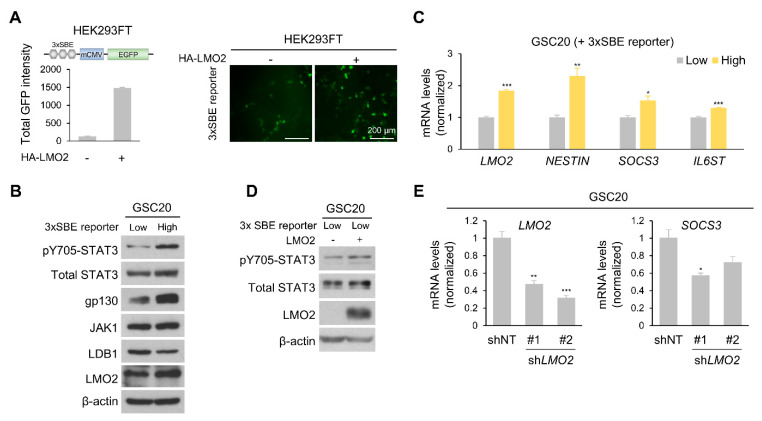
LMO2 and gp130 Are Key Factors for Activating STAT3 Signaling. (**A**) HEK293FT cells were transfected with the 3× STAT3 binding element (SBE) reporter vector, together with an expression vector encoding control or LMO2 (left panel). Representative fluorescence images of 3× SBE reporter system-induced U87MG cells transduced with either LMO2 or control expressing lentivirus (right panel). (**B**) Cell lysates of GSC20 cells (3× SEB reporter-High and -Low) transduced with either control or LMO2-expressing lentiviruses were immunoblotted with antibodies specific for pY705-STAT3, total STAT3, gp130, JAK1, LDB1, LMO2, and β-actin. (**C**) The mRNA levels of the indicated genes in 3× SBE reporter-High and -Low GSC20 cells were determined by real-time PCR. Data are expressed as mean ± SEM. The two-tailed Student’s *t*-test was used to analyze the statistical significance between each group (*n* = 3 for each group). * *p* < 0.05, ** *p* < 0.01, *** *p* < 0.001. (**D**) Cell lysates of GSC20 cells (3× SEB reporter-Low) transduced with either control or LMO2-expressing lentiviruses were immunoblotted with antibodies specific for pY705-STAT3, total STAT3, LMO2, and β-actin. (**E**) mRNA levels of the indicated genes in GSC20 cells transduced with either *LMO2* shRNA- or non-target shRNA-expressing lentiviruses were determined by real-time PCR. Data are expressed as mean ± SEM. The two-tailed Student’s *t*-test was used to analyze the statistical significance between each group (*n* = 3 for each group). * *p* < 0.05, ** *p* < 0.01, *** *p* < 0.001.

Embryonic stem cells and CSCs exhibit heterogeneity under in vitro culture conditions [39]. We hypothesized that the level of STAT3 activation varies in GSCs and correlates with LMO2. First, we constructed a STAT3 binding element (SBE) GFP reporter vector (3× SBE reporter) to detect STAT3 activation by the fluorescent signal intensity and transfected HEK293FT cells with a 3× SBE reporter vector in the presence or absence of the LMO2 expression vector. We observed a marked increase in STAT3 activation following LMO2 overexpression (Figure 3A). Next, we established a stable cell line of GSC20 cells expressing the 3× SBE reporter vector and sorted the cells into 3× SBE reporter-High and -Low cells. Immunoblot analysis showed that levels of pY705-STAT3, gp130, and LMO2 were higher in GFP-high GSC20 cells than in GFP-low GSC20 cells (Figure 3B). In addition, mRNA levels of *LMO2*, GSC marker (*NESTIN*), STAT3 target gene (*SOCS3*), and *GP130* increased in GFP-high GSC20 cells (Figure 3C). Transfection of LMO2 in GFP-low GSC20 cells led to an increase in pY705-STAT3 expression (Figure 3D). The shRNA-mediated depletion of LMO2 in GFP-high GSC20 cells decreased *SOCS3* mRNA expression (Figure 3E). Collectively, these data suggest that LMO2 plays a crucial role in STAT3 activation in GSCs.

### 3.4. ID1, A Downstream Target Gene of LMO2-STAT3 Signaling, Controls GSC Sphere Formation and Migration Abilities

**Figure 4:**
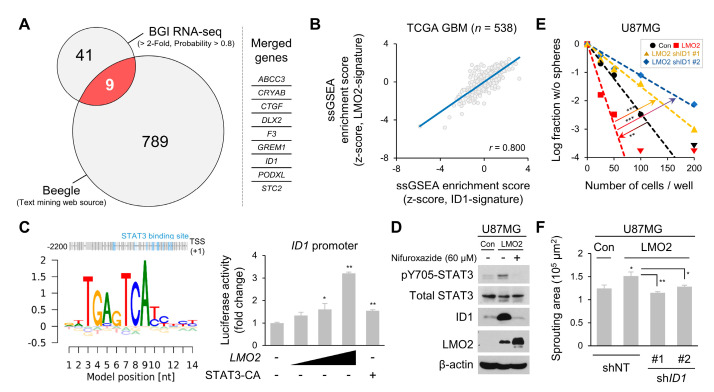
ID1, which is Elevated by LMO2-STAT3 Axis, Regulates Glioma Stem Cell (GSC) Properties. (**A**) Venn diagram showing overlapping genes between the 2-fold upregulated genes identified in the U87MG-LMO2 and glioblastoma-related genes searched by literature-based mining tool, Beegle [28] (https://beegle.esat.kuleuven.be/; search term: “glioblastoma”, accessed on 13 October 2019). Red: Merged genes. (**B**) Single-sample gene set enrichment analysis (ssGSEA) showed a positive correlation between LMO2-signature and ID1-signature in The Cancer Genome Atlas Glioblastoma (TCGA GBM) database. (**C**) Overview of binding sites for STAT3 in the promoter region 2200 bases upstream of the *ID1* transcription start site (TSS, +1). HEK293FT cells were transfected with the *ID1* promoter-driven luciferase reporter vector, together with an expression vector encoding constitutively active STAT3 and LMO2. Rectangle indicates increasing doses of the LMO2 expressing vector. Data are expressed as mean ± SEM. The two-tailed Student’s *t*-test was used to analyze the statistical significance between each group (*n* = 3 for each group). * *p* < 0.05, ** *p* < 0.01. (**D**) U87MG cells were transduced with either control or LMO2-expressing lentiviruses and exposed to nifuroxazide (60 µM) for 12 h. Cell lysates were immunoblotted with antibodies specific for pY705-STAT3, total STAT3, ID1, LMO2, and β-actin. (**E**) The tumorsphere forming ability of U87MG-control, U87MG-LMO2, and U87MG-LMO2-sh*ID1* cells was examined using the limiting dilution assay. The two-tailed Student’s *t*-test was used to analyze the statistical significance between each group (*n* = 24 for each group). ** *p* < 0.01, *** *p* < 0.001 (*n* = 24). (**F**) Quantification of the spheroid sprouting area in U87MG-LMO2 cells transduced with either *ID1* shRNA- or non-target shRNA-expressing lentivirus. Data are expressed as mean ± SEM. The two-tailed Student’s *t*-test was used to analyze the statistical significance between each group (*n* = 6 for each group). * *p* < 0.05, ** *p* < 0.01.

We performed RNA sequencing (RNA-seq) analysis using U87MG-control and U87MG-LMO2 cells to examine changes in gene expression by LMO2-STAT3 signaling in GBM. A total of 50 genes were significantly upregulated in U87MG-LMO2 cells. Among these genes, we found *ID1*, a master regulator of CSCs, as one of the LMO2-STAT3 signaling targets, using a literature-based mining tool [28,40] (Figure 4A). In addition, ssGSEA data showed that the LMO2-signature and ID1-signature were positively correlated in the TCGA GBM dataset (Figure 4B). To validate whether *ID1* is a downstream target of LMO2-STAT3 signaling, we developed a luciferase reporter gene construct containing a 2.2 kb upstream region of the human *ID1* promoter with STAT3 binding motifs. The reporter gene assay revealed that the constitutively active form of STAT3 increased *ID1* promoter activity; LMO2 also increased *ID1* promoter activity in a dose-dependent manner (Figure 4C). We further evaluated the effect of LMO2 or STAT3 inhibition on ID1 expression in LMO2-overexpressing non-GSCs. STAT3 inhibition by nifuroxazide inhibited the expression of ID1 [41] (Figure 4D).

ID1 has been shown to regulate GSC self-renewal via sonic hedgehog and Wnt signaling [42]. Therefore, we examined whether the LMO2-mediated increase in ID1 expression is involved in the stemness properties of GBM cells. We performed an in vitro limiting dilution assay and found that U87MG-LMO2 cells promoted sphere-forming activity, which was attenuated by shRNA-mediated *ID1* depletion in these cells (Figure 4E). These results suggest that ID1 is involved in the LMO2-mediated self-renewal of GBM cells. Next, we examined the stemness-related genes in U87MG-LMO2 cells transduced with *ID1*-shRNA or control-shRNA [14,43]. The increased *MYC* and *NESTIN* levels in U87MG-LMO2 cells were reduced by *ID1* depletion (Supplementary Figure S3A). In addition, 3× SBE reporter-High GSC20 cells had higher *ID1* mRNA levels than GFP-low GSC20 cells, and *LMO2* depletion in GFP-high GSC20 cells resulted in a decrease in *ID1* mRNA expression level (Supplementary Figure S3B).

A previous study demonstrated that ID1 promotes tumor invasiveness and migration [44]. Therefore, we examined whether the LMO2-mediated increase in ID1 expression is involved in the migration of GBM cells. We performed the spheroid sprouting assay and analyzed the sprouting area 3 h after seeding to exclude proliferation induced by LMO2. The results showed that U87MG-LMO2 cells expanded sprouting areas, and *ID1* knockdown restrained the sprouting capacity of U87MG-LMO2 cells (Figure 4F). These results suggest that LMO2-driven ID1 is involved in tumor migration, which is one of the worst prognostic markers in GBM [45]. Taken together, these results indicate that ID1 induced by the LMO2-STAT3 signaling axis regulates GSC properties and migratory ability.

## 4. Discussion

LMO2 was originally identified as a transcriptional adaptor for hematopoiesis and angiogenesis [19,20]. Meanwhile, a study has shown that cytoplasmic LMO2 plays a role in cellular signaling, such as the Wnt signaling pathway in breast and colorectal cancer models [25]. In addition, it is known that LMO4 interacts with STAT3 upstream effectors—JAKs and gp130 [46]. However, using TCGA databases, we found that the LMO4 & STAT3-signature did not have a prognostic value in 13 tumor types [47] (Supplementary Figure S3C). In cutaneous skin carcinoma, the level of LMO4 & STAT3-signature is correlated with a good prognosis (Supplementary Figure S3D).

Here, we propose that the interaction between LMO2 and STAT3 upstream effectors plays a key role in STAT3 activation in GSCs. Our results show that (1) LMO2 interacts with JAKs, gp130, and LDB1 in the cytoplasm; (2) the cytoplasmic LMO2-LDB1-gp130-JAKs complexes are present in GSCs but not in non-GSCs; (3) this interaction increases STAT3 activity; and (4) LMO2 elevates ID1 levels via STAT3 signaling, which is crucial for tumor aggressiveness. Thus, these results suggest a novel mechanism by which the cytoplasmic LMO2-LDB1 complex controls STAT3 activity.

Cancer heterogeneity is a major obstacle in understanding cancer and developing clinical strategies [48]. Notably, CSCs have been identified as a significant cause of cancer dynamics [49]. CSCs share some properties with NSCs, such as self-renewal, differentiation into various cell types to form a heterogeneous population, and residence in a stem cell niche that maintains CSCs in a stem cell statement [50]. However, the more aggressive the CSCs, the more independent the niche [51]. STAT3 signaling is essential for stemness in NSCs and CSCs [41,52,53]. Our data suggest that the cytoplasmic LMO2-LDB1 complex enhances STAT3 activity for the maintenance of CSC traits. Thus, it is likely that the specific control of STAT3 activity in CSCs can be achieved by targeting the LMO2-LDB1 complex.

Genetically identical cells, even within the same tissue, have a different intracellular context [54]. During angiogenesis, nuclear LMO2 enables differential regulation of the expression of angiogenic factors depending on the change in its binding partners [55,56]. Similar to the functional regulation of LMO2 in the nucleus, we found that the oncogenic function of cytoplasmic LMO2 is dependent on the presence of its interacting protein partners. For example, T-ALL cells, such as TF-1 cells, have high levels of LMO2 and its binding factor, but low levels of gp130. As a result, STAT3 activation is not observed. Taken together, our findings demonstrate that the functional interaction between the cytoplasmic LMO2-LDB1 complex and STAT3 upstream effectors is critical for maintaining GSC properties, and LMO2 acts as a new cytoplasmic adaptor for activating the oncogenic signaling cascade.

## 5. Conclusions

In this study, we addressed a novel mechanism by which the cytoplasmic LMO2 complex regulates STAT3 signaling. We suggested that the role of LMO2 in the cytoplasm rather than in the nucleus is related to the malignancy of brain tumors. However, in this study, it was not elucidated in what form the gp130-JAK1/2 receptor complex, additionally composed of LMO2 and LDB1, maintains itself. In addition, further investigation will be needed on whether these cytoplasmic LMO2-LDB1 complexes are actually found in in vivo mouse models or in patient tissues.

## Data Availability

The RNA sequencing data are available in the GEO repository (GSE182670 and GSE182169). LC-MS/MS data are available in PRIDE proteomics (PXD028254). Any other relevant data is available from the corresponding author (hg-kim@korea.ac.kr) upon request.

## Supplementary Materials

The following supporting information can be downloaded at: https://www.mdpi.com/article/10.3390/cells11132031/s1, Figure S1: LMO2 Is a Factor Which Is Associated with STAT3 Signaling in Glioma Stem Cells (GSCs); Figure S2: The LMO2-LDB1 Complex Binds with STAT3 Upstream Effectors. Figure S3: ID1 upregulated by LMO2-STAT3 signaling is related to MYC and NESTIN.; Figure S4: Uncropped western blot images of Figure 1; Figure S5: Uncropped western blot images of Figure 2; Figure S6: Uncropped western blot images of Figure 3; Figure S7: Uncropped western blot images of Figure 4; Figure S8: Uncropped western blot images of Figure S1; Figure S9: Uncropped western blot images of Figure S2; Supplementary Resources Table: Information of reagents and resource.
