# Supplementary material for: Cytoplasmic LMO2-LDB1 Complex Activates STAT3 Signaling through Interaction with gp130-JAK in Glioma Stem Cells
Source: Cells. 2022 Jun 26;11(13):2031. doi: 10.3390/cells11132031 (PMC9265747; doi:10.3390/cells11132031)
Supplement: Supplementary file 1 [file cells-11-02031-s001.zip › cells-1780642-supplementary.pdf]

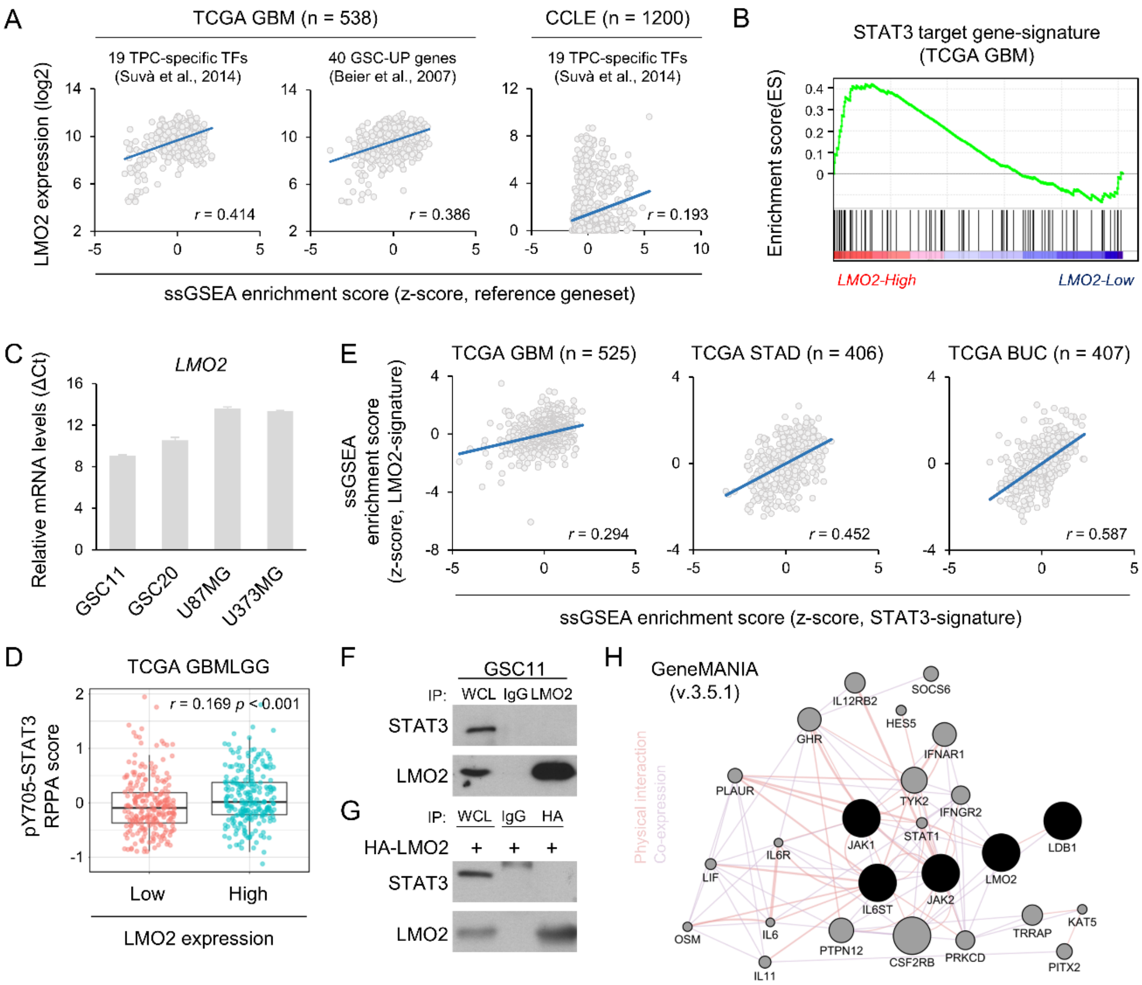

Supplementary Figure S1. LMO2 Is a Factor Which Is Associated with STAT3 Signaling in Glioma Stem Cells (GSCs). Single-sample gene set enrichment analysis (ssGSEA) showed a positive correlation between the expression of *LMO2* and each gene set (19 tumor propagating cells (TPC) –specific transcription factors (TFs) and 40 GSC-UP genes) in The Cancer Genome Atlas Glioblastoma (TCGA GBM) and Cancer Cell Line Encyclopedia (CCLE) **(A)**. Gene set enrichment analysis (GSEA) demonstrating enrichment of STAT3 activation target geneset in *LMO2*-High patients with GBM **(B)**. mRNA expression levels of *LMO2* in GSCs (GSC11 and GSC20) and non-GSCs (U87MG and U373MG) **(C)**. Reverse phase protein array showing a positive correlation between *LMO2* levels and pY705-STAT3 levels in the TCGA GBM Low grade glioma (LGG) database **(D)**. ssGSEA showing a positive correlation between LMO2-signature and STAT3-signature in TCGA GBM, TCGA Stomach adenocarcinoma (STAD), and TCGA Bladder urothelial carcinoma (BUC) database **(E)**. Endogenous co-immunoprecipitation of LMO2 in GSC11. Whole-cell lysates (WCLs, lane 1) or immunoprecipitates generated with LMO2 antibody (lane 3) or a control IgG antibody (lane 2) were immunoblotted with the indicated antibodies **(F)**. Co-IP of HA-LMO2 in HEK293FT cells HEK293FT cells were transfected with an expression vector encoding HA-LMO2. IgG is a control antibody for Co-IP. WCLs (lane 1) or immunoprecipitates generated with HA antibody (lane 3) or a control IgG antibody (lane 2) were immunoblotted with the indicated antibodies **(G)**. GeneMANIA shows factors that are capable of binding to LMO2, based on the results reported to date (*JAK1*, *JAK2*, *IL6ST* = STAT3 upstream effectors) **(H)**.

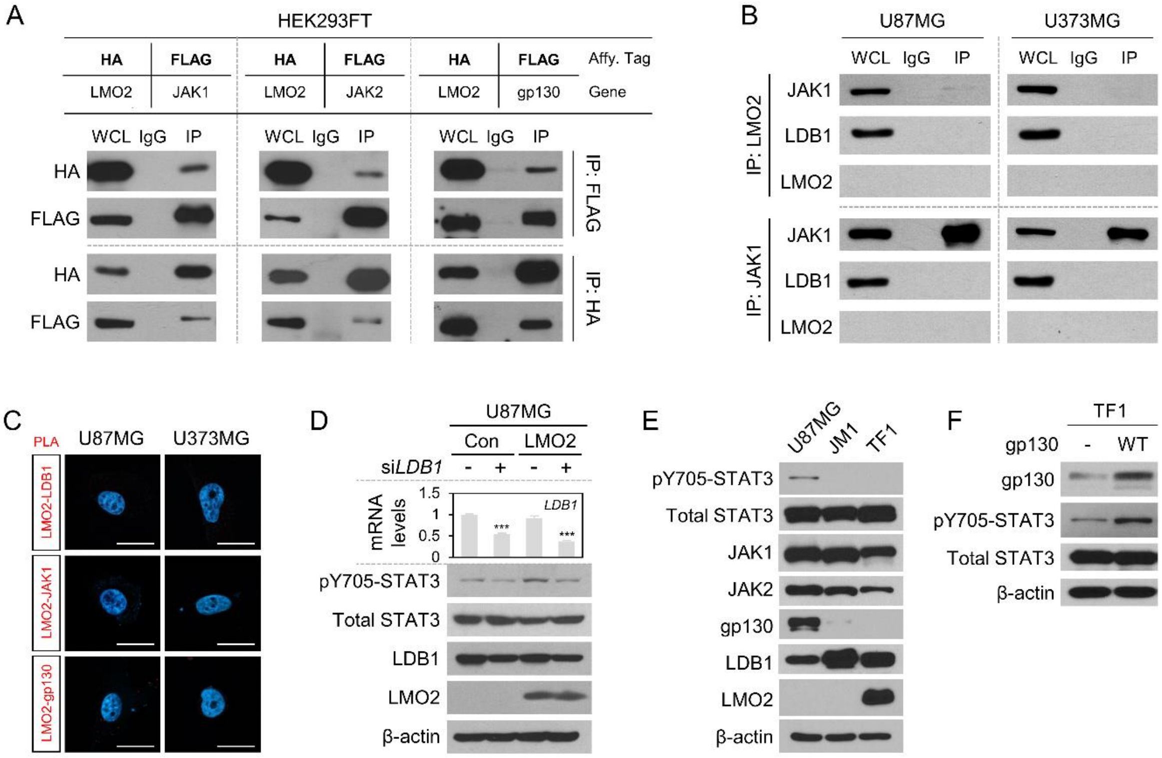

Supplementary Figure S2. The LMO2-LDB1 Complex Binds with STAT3 Upstream Effectors. Co-immunoprecipitation (Co-IP) of LMO2, JAK1, JAK2, and gp130 in HEK293FT cells. HEK293FT cells were transfected with expression vectors encoding HA-LMO2 together with FLAG-JAK1, FLAG-JAK2, or FLAG-gp130. IgG is a control antibody for Co-IP. Whole cell lysates (WCLs, lane 1) or immunoprecipitates generated with HA, FLAG (lane 3), or a control IgG antibody (lane 2) were immunoblotted with the indicated antibodies **(A)**. Co-IP of LMO2 and JAK1 in non-GSCs (U87MG and U373MG). IgG is a control antibody for Co-IP. WCLs (lane 1) or immunoprecipitates generated with the indicated antibodies (lane 3) or a control IgG antibody (lane 2) were immunoblotted with the indicated antibodies **(B)**. Proximity ligation assay in non-Glioma stem cells (U87MG and U373MG). The cells were stained with the indicated antibodies. Representative images are presented. Nuclei were stained with DAPI. Red dots indicate the protein interaction signals. Scale bar: 20 μm. **(C)**. mRNA expression levels of *LDB1* and its protein expression in U87MG cells expressing control vector or LMO2 overexpression vector-transfected with either non-target siRNA or si*LDB1* (10 nM) for 48 h. Cell lysates were immunoblotted with antibodies specific for pY705-STAT3, total STAT3, LDB1, LMO2, and β-actin **(D)**. Data are expressed as mean ± SEM. The two-tailed Student's *t*-test was used to analyze the statistical significance between each group (n = 3 for each group). ***P < .001. Cell lysates from U87MG, JM1, and TF-1 cells were immunoblotted with the indicated antibodies **(E)**. Cell lysates from TF-1 cells transduced with the control vector and gp130 overexpression vector were immunoblotted with the indicated antibodies **(F)**.

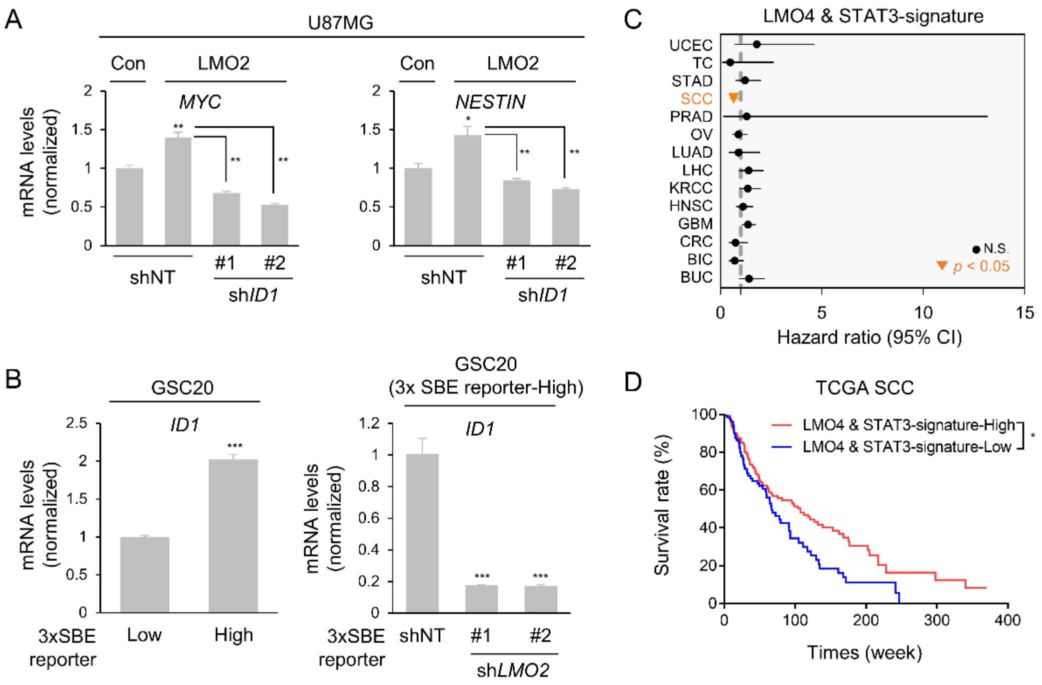

Supplementary Figure S3. ID1 upregulated by LMO2-STAT3 signaling is related to *MYC* and *NESTIN*. mRNA expression levels of *MYC* and *NESTIN* in U87MG cells expressing control vector or LMO2 overexpression vector-transfected with either non-target siRNA or si*ID1* (10 nM) for 48 h **(A)**. Data are expressed as mean ± SEM. The two-tailed Student's *t*-test was used to analyze the statistical significance between each group (n = 3 for each group). **P* < .05, ***P* < .01. The mRNA expression of *ID1* in 3x STAT3 binding element (SBE) reporter-High and-Low GSC20 cells was determined by real-time PCR (left panel). Data are expressed as mean ± SEM (n = 3). ****P* < .001. Analysis of 3x SBE reporter-High cells transduced with either *LMO2* shRNA- or non-target shRNA-expressing lentivirus was carried out using real-time PCR (right panel) **(B)**. Data are expressed as mean ± SEM. The two-tailed Student's *t*-test was used to analyze the statistical significance between each group (n = 3 for each group). ****P* < .001. Hazard ratio analysis using LMO4 & STAT3-signature in the pan-cancer dataset. (UCEC; uterine corpus endometrial carcinoma, TC; thyroid carcinoma, STAD; stomach adenocarcinoma, SCC; skin cutaneous carcinoma, PRAD; prostate adenocarcinoma, OV; ovarian serous cystadenocarcinoma, LUAD; lung adenocarcinoma, LHC; liver hepatocellular carcinoma, KRCC; kidney renal clear cell carcinoma, HNSC; head and neck squamous cell carcinoma, GBM; glioblastoma, CRC; colorectal adenocarcinoma, BIC; breast invasive carcinoma, BUC; bladder urothelial carcinoma). Horizontal bars represent 95% CIs of hazard ratios. Orange lines and dots represent tumors with statistically significant hazard ratios **(C)**. Kaplan-Meier survival analysis of patients with skin cutaneous carcinoma based on LMO4 & STAT3-signature levels. The log-rank Mantel-Cox test was used to analyze the statistical significance between overall survival of the patients with LMO4 & STAT3-signature-High (n = 289) and-Low (n = 290) **(D)**. **P* < .05.

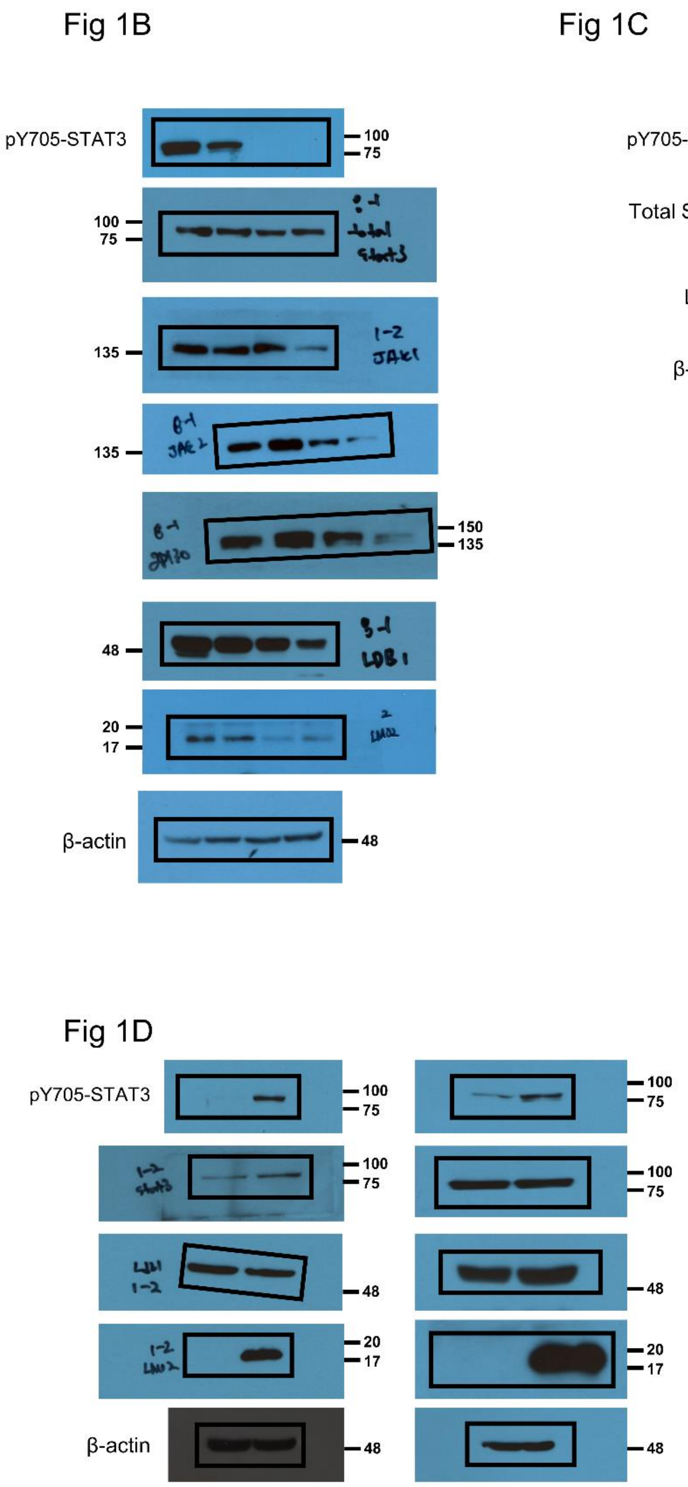

Supplementary Figure S4. Uncropped western blot images of Figure 1.

Fig 2B

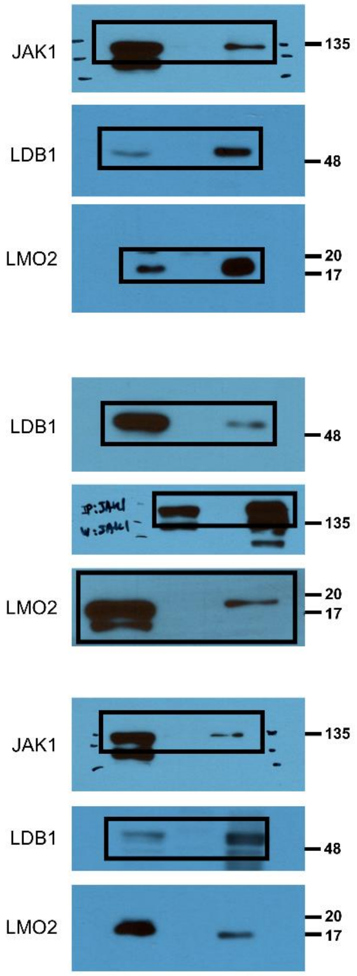

Fig 2C

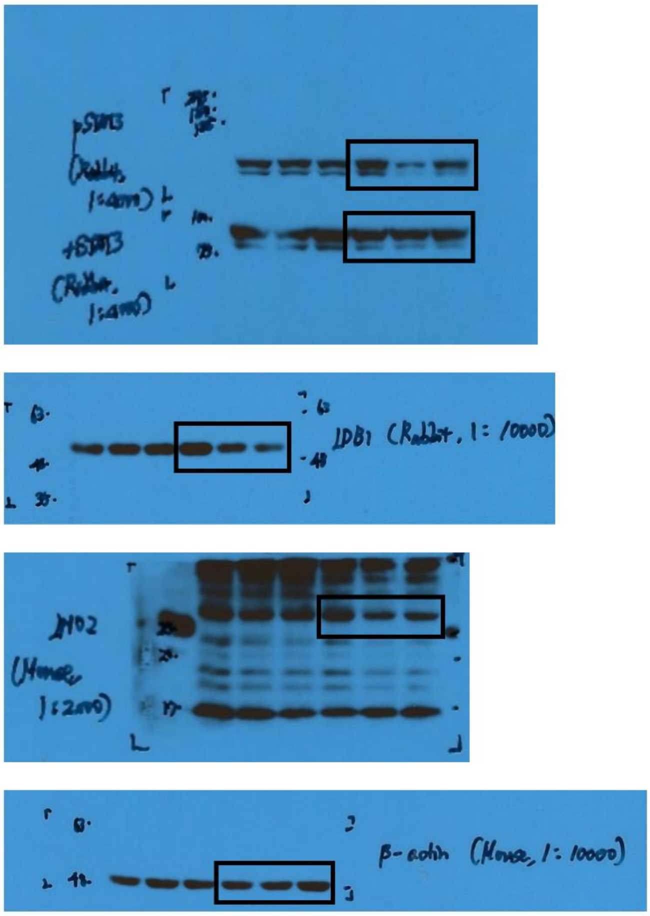

Fig 2E

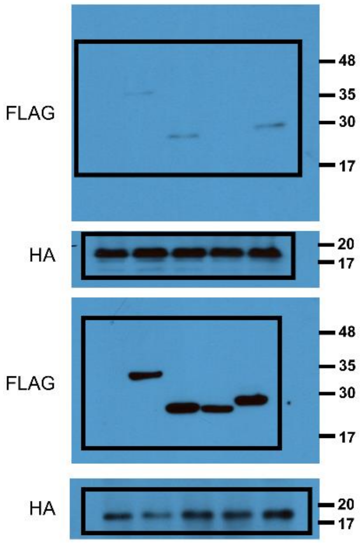

Fig 2F

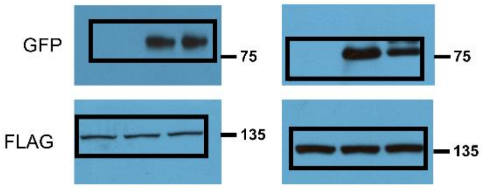

Fig 2G

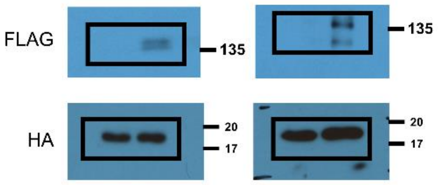

Supplementary Figure S5 Uncropped western blot images of Figure 2.

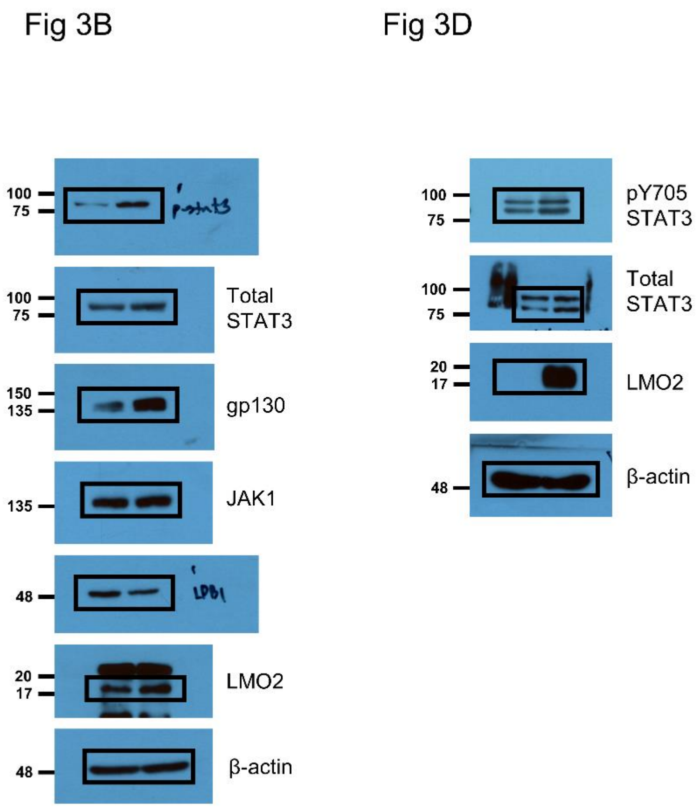

Supplementary Figure S6. Uncropped western blot images of Figure 3.

Fig 4D

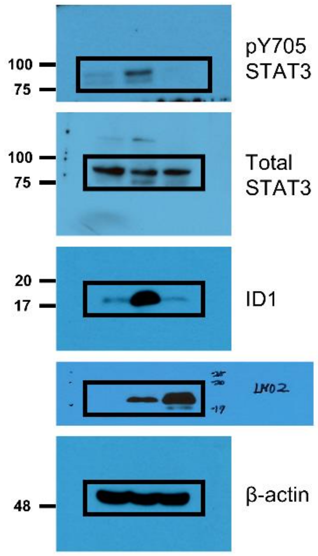

Supplementary Figure S7. Uncropped western blot images of Figure 4.

Fig S1F

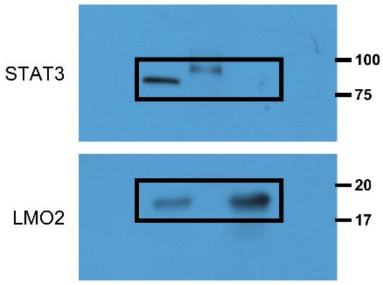

Fig S1G

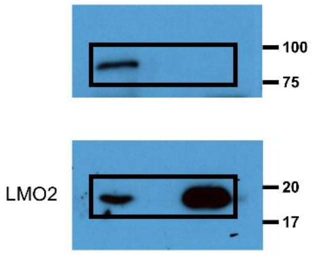

Supplementary Figure S8. Uncropped western blot images of Figure S1.

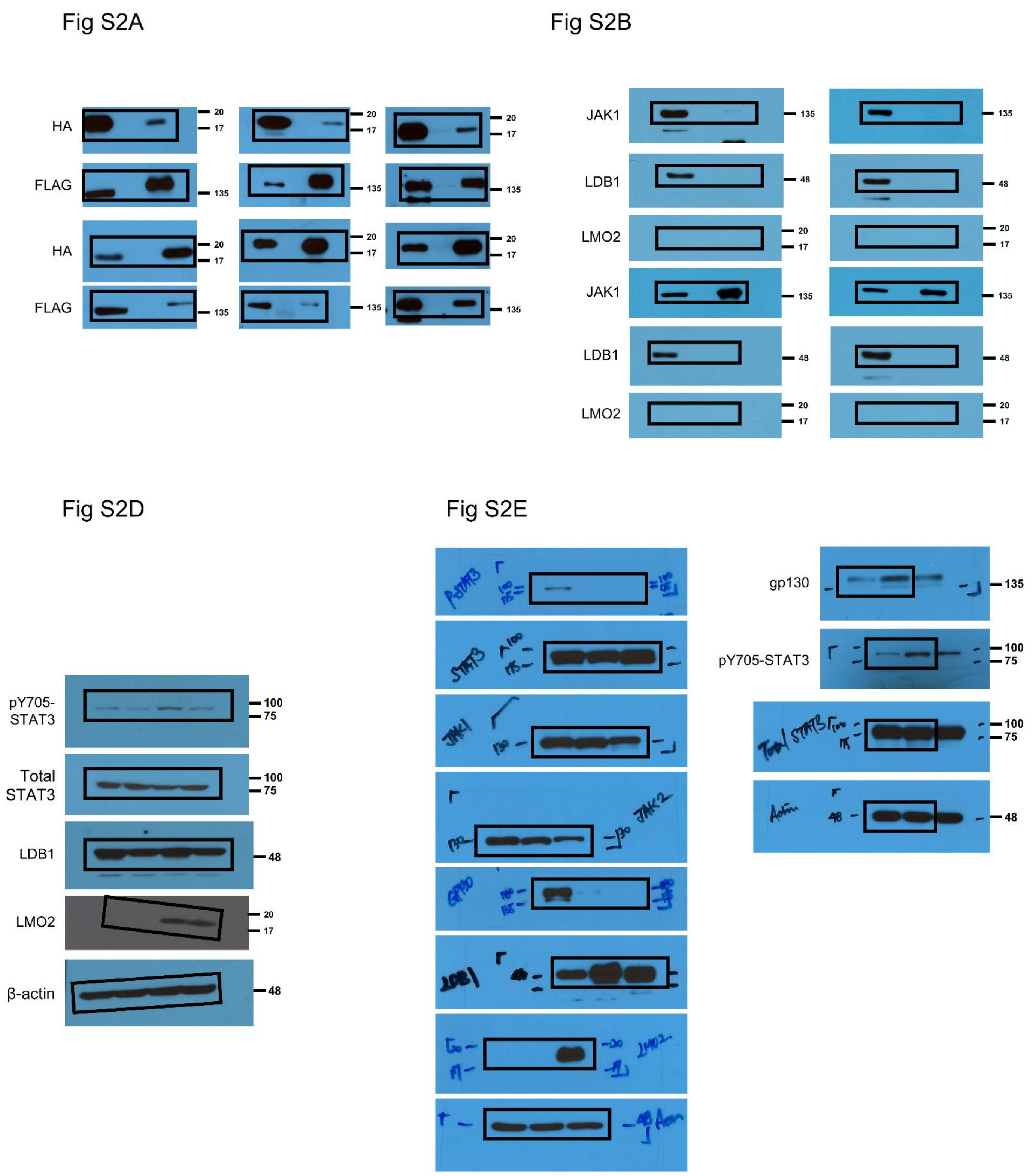

Supplementary Figure S9. Uncropped western blot images of Figure S2.

Supplementary Resources Table.

REAGENT or RESOURCE	SOURCE	IDENTIFIER
Antibodies		
α-LMO2	R&D systems	Cat# AF2726; RRID:AB_2249968
α-LDB1	Abcam	Cat# ab96799; RRID:AB_10679400
α-JAK1	Cell Signaling	Cat# 50996; RRID:AB_2716281
α-JAK2	Cell Signaling	Cat# 3230; RRID:AB_2128522
α-GP130	Santa Cruz Biotechnology	Cat# sc-656; RRID:AB_631591
α-GP130	Abcam	Cat# ab226346
α-β-ACTIN	Santa Cruz Biotechnology	Cat# sc-47778; RRID:AB_626632
α-pY705-STAT3	Cell Signaling	Cat# 9131; RRID:AB_331586
α-Total STAT3	Cell Signaling	Cat# 4904
α-ID1	Biocheck	Cat# BCH-1/195-14
α-HA	Cell Signaling	Cat# 3724
α-HA	Sigma	Cat# H9658
α-FLAG	Sigma	Cat# F7425; RRID:AB_439687
Normal mouse IgG	Santa Cruz Biotechnology	Cat# sc-2025
Normal rabbit IgG	Santa Cruz Biotechnology	Cat# sc-2027
Goat anti-rabbit IgG (H+L) Secondary antibody, HRP	Thermo Fisher	Cat# 31460
Goat anti-mouse IgG (H+L) Secondary antibody, HRP	Thermo Fisher	Cat# 31430
Mouse anti-goat IgG (H+L) Secondary antibody, HRP	Thermo Fisher	Cat# 31400
Bacterial and Virus Strains		
XL1-Blue SuperCompetent Cells	Agilent Technologies	Cat# 200236
Chemicals, Peptides, and Recombinant Proteins		
Fetal Bovine Serum	HyClone	Cat# SH30919.03
Penicillin-Streptomycin Solution	HyClone	Cat# SV30010
L-glutamine	HyClone	Cat# SH30034.01
Trypsin	HyClone	Cat# SH30042.01
Accutase solution	Sigma	Cat# A6964
DMEM/High Glucose	HyClone	Cat# SH30243.01
DMEM/F-12	HyClone	Cat# SH30023.01
RPMI-1640	HyClone	Cat# SH30027.01
B27 supplement	Invitrogen	Cat# 17504-044
Epidermal growth factor	R&D systems	Cat# 236-EG
Gentamicin Sulfate	Cellgro	Cat# MT-61-098-RF
Recombinant Human GM-CSF Protein	R&D systems	Cat# 215-GM-010
DNase I	Thermo Fisher	Cat# EN0525
RevertAid First Strand cDNA synthesis Kit	Thermo Fisher	Cat# K1622
Pierce IP lysis buffer	Thermo Fisher	Cat# 87787
cOmplete, Mini protease inhibitor cocktail	Roche	Cat# 11836153001
NuPAGE LDS Sample Buffer (4X)	Invitrogen	Cat# NP0007
Immobilon-P PVDF Membrane	Millipore	Cat# IPVH00010
PicoEPD Western reagent	Elpis Biotech	Cat# EBP-1073
RIPA buffer	LPS Solution	Cat# CBR002
BD Matrigel matrix	BD Biosciences	Cat# 354234
4’,6-diamidino-2-phenylindole	Sigma	Cat# D9542
Protein A agarose	Thermo Fisher	Cat# 20334
Protein G agarose	Thermo Fisher	Cat# 20339
QIAzol Lysis Reagent	QIAGEN	Cat# 79306

LipoJet™ In Vitro Transfection Kit (Ver. II)	SignaGen Laboratories	Cat# SL100468
ScreenFect A	Wako Puro Chemical	Cat# 299-73203
Lenti-X™ Concentrator	Clontech	Cat# 631231
Hexadimethrine bromide	Sigma	Cat# H9268
Phenylmethylsufonyl fluoride	Roche	Cat# 10837091001
Nifuroxazide	Sigma	Cat# 481984
Critical Commercial Assays		
TB Green® Premix Ex Taq™	Takara Bio Inc.	Cat# RR420A
Dual-Glo™ Luciferase Assay kit	Promega	Cat# E2940
Duolink™ In Situ Red Starter Kit Mouse/Rabbit	Sigma	Cat# DUO92101
Duolink™ In Situ PLA® Probe Anti-Goat PLUS	Sigma	Cat# DUO92003
RevertAid first-strand cDNA synthesis kit	Thermo Fisher	Cat# K1622
Experimental Models: Cell Lines		
U87MG	ATCC	Cat# HTB-14
U373MG	ATCC	Cat# HTB-17
HEK293FT	Thermo Fisher	Cat# R70007
JM1	ATCC	Cat# CRL-10423
TF-1	ATCC	Cat# CRL-2003
GSC11	Dr. Erik P. Sulman	N/A
GSC20	Dr. Erik P. Sulman	N/A
Oligonucleotides		
LDB1 RNAi targeting sequence: CUUGUAAAGCCCCCCCUG	Sigma	Cat# SASI-Hs02_00325821
LDB1 RNAi targeting sequence: CAAACUUGCCAGGGGAAG	Sigma	Cat# SASI-Hs01_00167846
Human *GAPDH* realtime PCR primer forward: CTACACTGAGCACCAGGTGGTCTC	This paper	N/A
Human *GAPDH* realtime PCR primer reverse: GATGGATACATGACAAGGTGCGGC	This paper	N/A
Human *NESTIN* realtime PCR primer forward: AACAGCGACGGAGGTCTCTA	This paper	N/A
Human *NESTIN* realtime PCR primer reverse: TTCTCTTGTCCCGCAGACTT	This paper	N/A
Human *SOCS3* realtime PCR primer forward: CCTGCGCCTCAAGACCTTC	This paper	N/A
Human *SOCS3* realtime PCR primer reverse: GTCACTGCGCTCCAGTAGAA	This paper	N/A
Human *IL6ST* realtime PCR primer forward: CATAGTCGTGCCTGTGTGCT	This paper	N/A
Human *IL6ST* realtime PCR primer reverse: GTGACCACTGGGCAATATGA	This paper	N/A
Human *LDB1* realtime PCR primer forward: TATCCGCCTACATACCTGGAG	This paper	N/A
Human *LDB1* realtime PCR primer reverse: GAAGCCGTTTGTTAAGCTCAAAT	This paper	N/A
Human *ID1* realtime PCR primer forward: AAACGTGCTGCTCTACGACA	This paper	N/A
Human *ID1* realtime PCR primer reverse: GATTCCGAGTTCAGCTCCAA	This paper	N/A
Human *MYC* realtime PCR primer forward: TCCTGGCAAAAGGTCAGAGT	This paper	N/A
Human *MYC* realtime PCR primer reverse: AGCTTTTGCTCCTCTGCTTG	This paper	N/A
Recombinant DNA		
pCDH-CMV-HA-FLAG-LMO2-puro	This paper	N/A
pcDNA3.1(+)-HA-LMO2-puro	This paper	N/A
pCMV-Tag2B-JAK1	This paper	N/A
pCMV-Tag2B-JAK2	This paper	N/A

pCMV-Tag2B-gp130	This paper	N/A
pcDNA3.1(+)-LIM1-GFP	This paper	N/A
pcDNA3.1(+)-LIM2-GFP	This paper	N/A
pcDNA3.1(+)-FLAG-FERM	This paper	N/A
pcDNA3.1(+)-FLAG-SH2	This paper	N/A
pcDNA3.1(+)-FLAG-JH2	This paper	N/A
pcDNA3.1(+)-FLAG-JH1	This paper	N/A
pcDNA3.1(+)-FLAG-gp130^{ID}	This paper	N/A
pLKO.1-puro-shNT	Addgene	Cat# 109012
pLKO.1-shLMO2 #128 targeting sequence: CCATAGTAACTGACATGATTA	Sigma	Cat# TRCN0000017128
pLKO.1-shLMO2 #129 targeting sequence: GCGGGTGAAAGACAAAGTGTA	Sigma	Cat# TRCN0000017129
pLKO.1-shID1 #1 targeting sequence: CCTACTAGTCACCAGAGACTT	This paper	N/A
pLKO.1-shID1 #2 targeting sequence: ACTCGGAATCCGAAGTTGGAA	This paper	N/A
pCDH-3xSBE-mCMV-EGFP	This paper	N/A
Software and Algorithms		
Image j	National Institutes of Health	https://imagej.nih.gov/ij/
Beegle	N/A	http://beegle.esat.kuleuven.be/
Contra v3	N/A	http://bioit2.irc.ugent.be/contra/v3/
MassMatrix	MassMatrix	https://massmatrix.bio/
Prism 6	Graphpad	https://www.graphpad.com
ELDA	N/A	https://bioinf.wehi.edu.au/software/elda
IncuCyte 2019B Rev2	Satorius	N/A
GeneMANIA v3.5.1	N/A	https://genemania.org
ZEN 3.1 (blue edition)	Carl Zeiss	https://zeiss.com
Deposited data		
LMO2 RNA-sequencing data	This paper	GSE182169
ID1 RNA-sequencing data	This paper	GSE182670
LMO2 binding protein LC-MS/MS data	This paper	PXD028254
